# Chloroform in Rheumatism

**Published:** 1853-10

**Authors:** J. Kirby

**Affiliations:** Ex-prof. of Physic in the College of Surgeons, Ireland


*Chloroform in Rheumatism.* By J. Kirby, L.L.D., L. C.D., Ex-prof. of Physic in the College of Surgeons, Ireland.—The following case is selected from some others of the same kind, as illustrating a kind of practice that we have seen carried out in several instances with marked benefit:—On the 3d instant, I was called to visit Mrs. K—, who is about 40, mother of several children, and now six months advanced in pregnancy. At the commencement of last summer, she was affected with a scrofulous abscess above Poupart's ligament at the left side, which was opened and discharged matter in a large quantity peculiar to that disease. By a residence in the country, seabathing, suitable exercise, and tonic remedies, the abscess grew less, and in time it cicatrized altogether, On her return to town, she again complained of abdominal uneasiness, the tumor formed anew, and it broke spontaneously, she in the mean time having no advice on the subject.

For the last fortnight, she has been confined to her bed, except when moved for purpose of convenience or comfort. She lies wholly on her right side, in consequenc of pain induced whenever she turns on her left. During this time she had "scarcely any sleep." At night she suffers "most excruciating and constant pains in her loins, over the sacrum, the coccyx, and the hips." These pains compel her to loud exclamations of distress, disturbing the whole house by her vociferations, so that her husband and attendants make many complaints of the want of repose. The appetite is wholly gone. She takes whey and tea principally and she loathes wine, porter, ale and beer. She sweats profusely at night; the bowels are regular; the urine scanty and lateritious: the pulse is very frequent and weak: she is much emaciated, and looks in fact like a person far advanced in hectic fever. The movements of her child are quick, lively and natural. The abscess discharges about a tablespoonful of rather healtny matter

From the success which attended the administration of chloroform in other cases, I was induced to make a trial of its remedial agency in the present instance, and I accordingly prescribed it in the following way:—

℞.—Chloroform. min. octo.

Aquæ ℥j;

Syrup. croci ʒj;

Ft. haustus. Mitte tres haustus tales. St. unum quartis horis.

℞.—Chloroform. drach. tres.
Aquæ unc. tres. M. Sig. for external use.

A sixth part of mixture was desired to be sprinkled on a folded handkerchief and laid on the parts severely affected, and these to be covered with a large sheet of gutta percha paper to prevent evaporation. Visiting her the next day, I was agreeably surprised to learn from her that all her torments had wholly disappeared after she had taken the first draught, and that the others had procured a good night's sleep.

The first application of the mixture was awkwardly applied, and she said it gave her much pain, and therefore she would not permit it to be used again. In consequence of a good night, she had some appetite, and enjoyed a glass of Calcavella wine. Two chloroform draughts were to be taken at night if the pains returned.

The husband has informed me that she was moved to the sofa at four o'clock yesterday, where she remained till twelve, and slept, as she was free from uneasiness. On her return to her bed, she was seized violently in the back and hips, but these pains were much alleviated by the two chloroform draughts.

She was now ordered to remain in her bed, to have the local application made once, and to take two draughts at night unconditionally, and a mixture composed of infusion of roses, with dilute sulphuric acid, was ordered with a view to restrain the nightly perspiration, to which she attributes much of her debility.

To-day, the 7th of the month, she reports that she used no medicines, feeling that she required none; however, she is mistaken, for her health is in a very precarions state in consequence of the discharge of matter from her groin and her pregnancy. To-day she goes two miles from town, and her case devolves on Dr. O'Brien, who met me in consultation, and who is to attend her in her confinement.—*Dublin Medical Press.*

